# A simple method to remove ticks, no tools needed

**DOI:** 10.1111/jdv.70145

**Published:** 2025-10-27

**Authors:** Danai Bucher, Olivier Gaide

**Affiliations:** ^1^ Department of Dermatology and Venereology Lausanne University Hospital Lausanne Switzerland

**Keywords:** Ixodes, removal, technique, ticks

Erythema chronicum migrans is a sign of cutaneous boreliosis, transmitted by tick bites (Figure 1). Live ticks can be removed without any tools or assistance. Indeed, a gentle finger is all that is needed to remove a tick. Place the finger on the tick, apply light pressure and make the tick rotate around its head, like a merry‐go‐round. After a series of turns, the tick will release its hold. This avoids leaving any part of the tick in the skin, as can happen when using a tweezer or similar. The method is rapid, efficient and painless. It can be performed easily on the field, in a private practice or in a hospital. It can be used on kids and adults. It is very cost‐efficient, certainly the cheapest method we know of. The single limitation of this technique is that it does not work on dead ticks. Please try it, film it like we did (Movie S1), and spread the info! Maybe get it on TikTok (pun intended).

**FIGURE 1:**
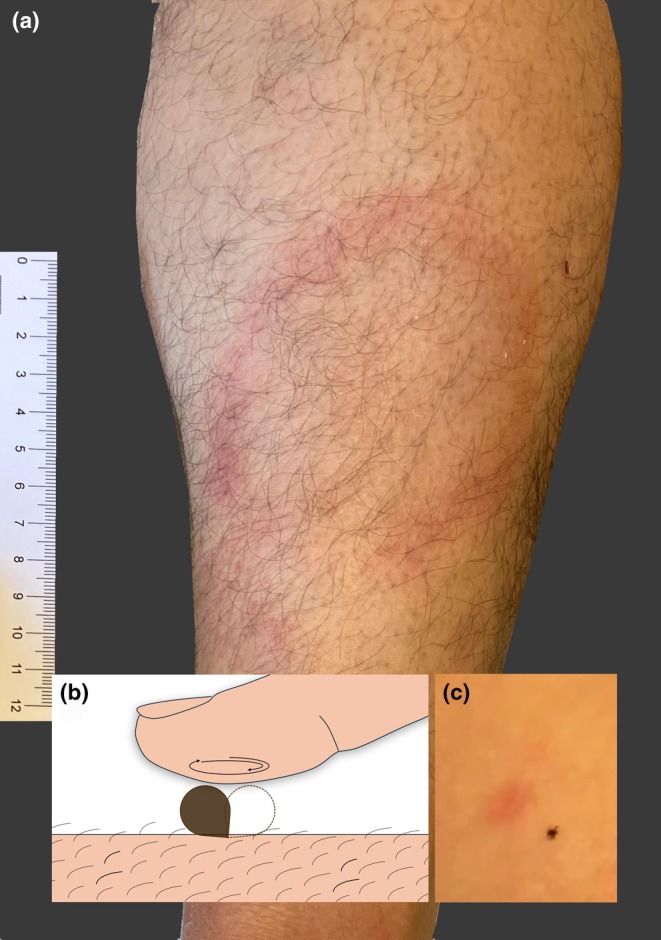
(a) Erythema chronicum migrans on the leg of a male patient. (b) Schematic drawing of a finger applying gentle pressure on a tick, then making spinning movements, forcing the tick's body to rotate around its biting‐hold. (c) Ixodes tick shown after it released its hold and walked away from the red erythematous macule where it bit the patient.

## FUNDING INFORMATION

The authors received no funding for this study.

## CONFLICT OF INTEREST STATEMENT

The authors have no conflict of interest to declare.

## ETHICAL APPROVAL

Our institution does not require ethical approval for reporting analysed pictures of individual cases.

## ETHICS STATEMENT

Patient consent was obtained for pictures and video. The ticks were euthanized ethically after removal.

## Supporting information


**Video S1.** Video showing live ticks releasing their skin hold after being rotated with a gentle pressure of the finger.

## Data Availability

The data that support the findings of this study are available from the corresponding author upon reasonable request. Unedited pictures and movies are available upon request.

